# Extending seasonal malaria chemoprevention to five cycles: a pilot study of feasibility and acceptability in Mangodara district, Burkina Faso

**DOI:** 10.1186/s12889-022-12741-9

**Published:** 2022-03-05

**Authors:** Adama Traore, Laura Donovan, Benoit Sawadogo, Charlotte Ward, Helen Smith, Christian Rassi, Helen Counihan, Johanna Johansson, Sol Richardson, Justin Ragnessi Savadogo, Kevin Baker

**Affiliations:** 1 Malaria Consortium, Ouagadougou, Burkina Faso; 2 Malaria Consortium, London, UK; 3 London School of Hygiene & Tropical Medicine, London, UK; 4 International Health Consulting Services Ltd, Hoylake, UK; 5 Malaria Consortium, Lomé, Togo; 6 Ministry of Health, Ouagadougou, Burkina Faso

**Keywords:** Seasonal malaria chemoprevention, Malaria, Burkina Faso, Acceptability, Feasibility

## Abstract

**Background:**

Seasonal malaria chemoprevention (SMC) involves administering antimalarial drugs at monthly intervals during the high malaria transmission period to children aged 3 to 59 months as recommended by the World Health Organization. Typically, a full SMC course is administered over four monthly cycles from July to October, coinciding with the rainy season. However, an analysis of rainfall patterns suggest that the malaria transmission season is longer and starting as early as June in the south of Burkina Faso, leading to a rise in cases prior to the first cycle. This study assessed the acceptability and feasibility of extending SMC from four to five cycles to coincide with the earlier rainy season in Mangodara health district.

**Methods:**

The mixed-methods study was conducted between July and November 2019. Quantitative data were collected through end-of-cycle and end-of-round household surveys to determine the effect of the additional cycle on the coverage of SMC in Mangodara. The data were then compared with 22 other districts where SMC was implemented by Malaria Consortium. Eight focus group discussions were conducted with caregivers and community distributors and 11 key informant interviews with community, programme and national-level stakeholders. These aimed to determine perceptions of the acceptability and feasibility of extending SMC to five cycles.

**Results:**

The extension was perceived as acceptable by caregivers, community distributors and stakeholders due to the positive impact on the health of children under five. However, many community distributors expressed concern over the feasibility, mainly due to the clash with farming activities in June. Stakeholders highlighted the need for more evidence on the impact of the additional cycle on parasite resistance prior to scale-up. End-of-cycle survey data showed no difference in coverage between five SMC cycles in Mangodara and four cycles in the 22 comparison districts.

**Conclusions:**

The additional cycle should begin early in the day in order to not coincide with the agricultural activities of community distributors. Continuous sensitisation at community level is critical for the sustainability of SMC and acceptance of an additional cycle, which should actively engage male caregivers. Providing additional support in proportion to the increased workload from a fifth cycle, including timely remuneration, is critical to avoid the demotivation of community distributors. Further studies are required to understand the effectiveness, including cost-effectiveness, of tailoring SMC according to the rainy season. Understanding the impact of an additional cycle on parasite resistance to SPAQ is critical to address key informants’ concerns around the deviation from the current four-cycle policy recommendation.

## Background

In 2019, 67% of all global deaths due to malaria occurred in children aged under five, with 272,000 deaths recorded and 94% of these occurring in sub-Saharan Africa [1]. Prevention of malaria remains a major public health issue with complex challenges such as the rise of drug and insecticide resistance, however the burden of malaria is progressively declining in most of sub-Saharan Africa [2]. In Burkina Faso, malaria remains endemic [3], and in 2018 it was the most common cause of medical consultations (41.3%) and death (16.4%) according to national data from health facilities [4].

To reduce morbidity and mortality from malaria, the World Health Organization (WHO) recommends Intermittent Preventive Treatment in infancy (IPTi), Intermittent Preventive Treatment of pregnant women (IPTp) and seasonal malaria chemoprevention (SMC) for children aged 3–59 months [5, 6]. SMC is recommended in the Sahel region where malaria transmission is highly seasonal, preventing three-quarters of all uncomplicated and severe malaria cases [7]. Each monthly administration involves providing a full course of sulfadoxine-pyrimethamine (SP) plus amodiaquine (AQ); one dose of SP and the first dose of AQ are given under the supervision of a community distributor (CD) as directly observed treatment (DOT) on Day 1, and the remaining two doses of AQ are given by the caregiver over the following two days. SMC drugs are typically administrated through a door-to-door strategy, with CDs being recruited through community leaders, to support the health facility in the drug distribution. They receive training on SMC drug administration and sensitisation towards malaria prevention. Typically, this approach starts at the beginning of the malaria transmission season, with a full course of SPAQ being administered at 1-month intervals (SMC cycle) up to a maximum of four cycles in a year (SMC round).

SMC has been implemented in Burkina Faso since 2014 [8] and evidence demonstrates its feasibility, cost-effectiveness and impact on malaria morbidity and mortality at scale [9, 10]. In 2019, approximately 15,000 infants (aged 3–11 months) and 15,000 young children (aged 12–59 months) received SMC in the health district of Mangodara [11]. Malaria is highly seasonal in Burkina Faso, where SMC has historically been implemented from July to October, with an estimated 60 percent of malaria cases occurring during these months. However, based on meteorological data and an analysis of seasonal rainfall patterns, there is evidence that the malaria transmission season now starts as early as June in the southern part of the country, resulting in a longer transmission period. This is when the number of malaria cases among children under five increases rapidly, before the start of the SMC drug distribution in July [12]. Data from the Surveillance National Information System (SNIS) of the Ministry of Health show that from 2016 to 2018, the highest number of malaria cases in children under five in the health district of Mangodara occurred in July [13].

There is a small body of evidence demonstrating the effectiveness of administering SMC over five months, rather than four, in areas of extended seasonal malaria transmission. A randomised clinical trial conducted in Ghana in 2016 found that five cycles of SMC administered during the peak of the malaria transmission season reduced the burden of malaria by around 50% [14]. A study in Senegal in 2011 found that SMC given over five months was feasible, well tolerated and effective in preventing malaria episodes, and reduced the prevalence of parasitaemia and anaemia [15]. Qualitative research on the acceptability of SMC drug delivery over five cycles indicates that caregivers perceive positive health effects in children who take more than four monthly doses of SMC [16]. Evidence of the feasibility and acceptability of extending SMC from four to five cycles could facilitate a change in SMC policy based on variations in malaria seasonality across the Sahel, and improve awareness and perceptions of caregivers towards SMC.

In 2019, Malaria Consortium supported SMC implementation in a total of 23 health districts in Burkina Faso. In this study, we assessed whether implementation of an additional SMC cycle (‘cycle 0’) to eligible children in Mangodara health district at the start of the rainy season in June, was feasible and acceptable to caregivers of children under five, community distributors and stakeholders at the district, regional and national level. We also assessed the effect of the additional cycle on the coverage of SMC in Mangodara, defined as coverage of Day 1 SPAQ, compared with the 22 other health districts where SMC was implemented with support from Malaria Consortium in the same month, in addition to the proportion of eligible children who received all cycles of SMC delivered during 2019.

## Methods

### Study design

This was a small-scale pilot implementation research study, conducted by Malaria Consortium in collaboration with the National Malaria Control Programme (NMCP), Burkina Faso. This study used a convergent mixed-methods approach, in which qualitative and quantitative data were collected and analysed during a similar timeframe. Quantitative data were collected through end-of-cycle (EoC) and end-of-round (EoR) household surveys, which are explained further below. Qualitative data were collected through focus group discussions (FGDs) and key informant interviews (KIIs).

### Study setting and population

The study was conducted in Mangodara health district in the south-west of Burkina Faso, where the region exhibits an extended rainy season and malaria transmission peaks between June to August, tailing off in November/December [17]. Mangodara has the highest malaria prevalence in the country; according to 2017–18 estimates [18], 39% of children under five are infected, compared with the national average of 17%. In our study, SMC cycles one to four were implemented in parallel with routine SMC administration from July to November 2019, with the additional cycle (‘cycle 0’) being implemented in June in Mangodara.

The study population comprised of 30,645 children aged 3–59 months residing in Mangodara health district who met the standard eligibility criteria for SMC, i.e. who did not receive SP or AQ in the last 28 days, did not have known allergies to SPAQ and did not have a fever. Caregivers of children who participated in the 2019 SMC campaign were also enrolled, along with CDs who participated in the five-cycle campaign in 2019, for the FGDs. Key informants at the community, programme and national-level were invited to take part in the KIIs.

### Data collection

Quantitative data were collected through four EoC household surveys; these are typically conducted following cycles 1, 2 and 3 using lot quality assurance sampling (LQAS) methodology to assess adherence to, and coverage of, Day 1 SPAQ among eligible children [19]. The survey enabled coverage in cycle 0 to be determined, along with a comparison of coverage between districts in the same month. These data helped to determine whether any difference in coverage may have implications for the generalisability of the results to districts outside Mangodara. Data were also collected through an EoR survey following completion of SMC cycle four, to evaluate coverage at the end of the annual campaign, including quality of program implementation. The survey protocol is described in detail elsewhere [20].

Data were collected by research assistants in French with mobile phones and transferred to an electronic data collection platform; Magpi. Data quality was ensured through a standard operating procedure (SOP) to standardise methods for data capture, along with the presence of a Malaria Consortium Project Coordinator to oversee the data collection process.

Specifically, data were collected to determine the difference in coverage (%) of children aged 3–59 months who received a full course of SMC in 2019 (five cycles) compared to 2018 (four cycles). Additionally, the coverage (%) of children aged 3–59 months who received each cycle of SMC in 2019 compared to 2018 was determined. The survey was designed to obtain a proportionally larger sample of respondents from Mangodara. Within each of the 22 health districts where Malaria Consortium implemented SMC in 2019, 11 villages were selected. This resulted in a total of 242 villages being surveyed, of which 66 were located in Mangodara and 176 were located in the other 22 districts, and the protocol aimed to achieve self-weighting samples of households with at least one eligible child aged 3–59 months. Villages were selected randomly with probability according to their size, and the protocol specified that a constant number of households [15] should be sampled per village. On average, across all districts, 16.8 households were sampled per village; this resulted in a total sample of 5,066 households. Of these, 4,999 had at least one SMC-eligible child and agreed to participate in the survey; 1,068 in Mangodara and 3,931 in the other 22 districts, of which 1,063 and 3,909 provided data on the coverage status of Day 1 SPAQ of a randomly-selected child, respectively.

Qualitative data were collected through separate FGDs with community distributors (*n* = 4) and caregivers of children aged under five (*n* = 4), resulting in a total of 8 FGDs. The selection of health facilities and villages was randomised; villages were each assigned a random number which was then chosen out of a box. Within the selected villages, the process was repeated to anonymously select the health facilities. Of the 26 health facilities in Mangodara, two were randomly selected and within these, respective CDs were identified and selected based on their availability and willingness to participate in consultation with the head of the facility. Those who participated in the implementation of the 2019 SMC campaign in Mangodara, including the additional cycle, were considered eligible for enrolment. A village in each of the health facility catchment areas was randomly selected and within each village, caregivers were recruited in consultation with their respective village leader through convenience sampling. Caregivers were deemed eligible to participate if they had a child who participated in the 2019 SMC campaign.

Each FGD was composed of 6–8 participants, and for both participant groups FGDs were split by gender to facilitate open discussion. However, due to a limited number of female CDs in a particular district, one FGD was mixed. All FGDs were held in Dioula, the local language most commonly spoken in Mangodara, and audio recorded using a digital recorder. Eleven KIIs were held with key stakeholders at various levels of the health system; supervisors of community distributors (*n* = 2), community leaders (*n* = 4) and national programme partners (*n* = 5) in the SMC programme. Interviews with community leaders were held in the villages and interviews with national stakeholders in their respective offices. FGDs and KIIs were conducted by research assistants and the Malaria Consortium Research Coordinator, respectively. Community-level key informants were identified and selected through convenience sampling, based on their availability and willingness to participate. Programme and national-level informants were identified and selected by the Malaria Consortium team based in Ouagadougou, on the basis of their role and experience of working on the SMC programme in Burkina Faso. Interviews were conducted in French, except those with the community leaders which were held in Dioula. The FGDs and KIIs aimed to elicit participant perceptions of the feasibility and acceptability of the additional SMC cycle.

### Data analysis

SPSS was used to analyse quantitative data to calculate the coverage of SMC among eligible children aged 3–59 months in each cycle based on caregiver reports. We then compared coverage between districts (between Mangodara and the 22 other districts) in the same month using the chi-square statistic, estimating *p*-values (without Yates’ correction) for significance of difference in coverage between districts. As a sensitivity analysis, we compared coverage by cycle in Mangodara versus the other 22 districts according to the order of the cycles (e.g. comparing the first cycle in Mangodara, cycle 0 in June, with the first cycle in the other 22 districts, cycle 1 in July).

In addition, we calculated the proportion of children by total number of cycles they received over the 2019 SMC round, and compared the proportions of children by numbers of cycles received in Mangodara with that in the other 22 districts.

Qualitative data from the FGDs and KIIs conducted in Dioula were first translated into French from the audio recording, and then transcribed into English. Data quality was ensured by checking a sample of the transcripts in French and English for translation accuracy through back-translation into Dioula. A thematic analysis was conducted using the software MAXQDA 2020; an initial coding frame was developed to code the transcripts, with codes collated and categorised into emerging themes. Summaries of each theme were reviewed and discussed by the whole team before final consolidation. Themes related to reactions to the five-cycle campaign, feasibility and acceptability of five cycles and views on future campaigns.

### Informed consent and ethical considerations

Prior to the EoC and EoR surveys being conducted, participants were given an information sheet and consent form to read, including information on the withdrawal of consent at any point, and given contact details of the Survey Coordinator. For illiterate participants, data collectors read the information sheet and consent form aloud in the local language, Dioula. Data collectors confirmed their consent to participate by ticking a box on the data collection platform, SurveyCTO.

Prior to the commencement of FGDs and KIIs, sensitisation meetings were held in each village. Participants included the district health official and head of the village to discuss the aims of the research, study process, and to obtain permission for the villagers to participate. Participants were allowed up to an hour to confirm their participation, and were guided through the information sheets provided to assist their decision. Participants of the study gave their consent to participate in the qualitative component by signing an informed consent form or marking their digital fingerprint if illiterate.

FGDs were conducted in a quiet and neutral space which was kept private for the duration of the discussion; for CDs, this was near their respective health facility.

To ensure participant confidentiality and data authenticity, audio files and transcriptions of FGDs and KIIs were kept in secure, locked cabinets, accessible to the Malaria Consortium Project Coordinator. Paper consent forms and FGD and KII topic guides were stored in opaque carriers at all times. Subject identifiers were not utilised on data forms or during FGDs or KIIs; a unique identification code was used to anonymise subject data e.g. 1001.

This study was approved by the Comité Institutionnel de Bioéthique of Centre National de Recherche et de Formation sur le Paludisme (Burkina Faso) on 1^st^ August 2019, reference: n°2019/000005/MS/SG/INSP/CNRFP/CIB.

## Results

### Quantitative results

Table 1 below shows the coverage per SMC cycle and proportion of children aged 3–59 months who received all five SMC cycles in Mangodara district and all four SMC cycles in the other 22 districts in 2019. Results of chi-squared tests for differences between Mangodara and the other 22 districts are shown.

**Table 1:** SMC coverage per cycle to eligible children in Mangodara compared to 22 other SMC districts

Variable	Mangodara District *n* = 1063 N (%)	22 Districts *n* = 3909 N (%)	χ2 value (df = 1)	*p*-value
Coverage of eligible children by cycle				
Cycle 0 (June 2019)	932 (87.67%)	N/A		
Cycle 1 (July)	956 (89.9%)	3419 (87.46%)	4.82	0.028
Cycle 2 (August)	998 (93.89%)	3471 (88.79%)	23.81	<0.001
Cycle 3 (September)	1005 (94.54%)	3483 (89.10%)	28.16	<0.001
Cycle 4 (October)	1009 (94.92%)	3760 (96.19%)	3.43	0.063
Coverage of eligible children by number of cycles				
No cycle	5 (0.47%)	22 (0.56%)	0.13	0.716
At least 1 cycle	1058 (99.53%)	3887 (99.43%)	0.13	0.716
At least 2 cycles	1034 (97.27%)	3637 (93.04%)	26.29	<0.001
At least 3 cycles	1006 (94.63%)	3556 (90.96%)	14.86	<0.001
At least 4 cycles	944 (88.81%)	3297 (84.34%)	13.26	<0.001
At least 5 cycles	872 (82.03%)	N/A		

Coverage of Day 1 SPAQ among eligible children was 87.67% in Mangodara in cycle 0. Although this proportion was marginally lower than in cycles 1–4, coverage in cycle 1 in the 22 other health districts was also lower than in subsequent cycles.

The results of the chi-squared tests show that coverage of Day 1 SPAQ was significantly higher in Mangodara than in the 22 other districts in cycles 1, 2 and 3 but significantly lower in cycle 4. The results of the sensitivity analysis showed that the coverage of eligible children in Mangodara was significantly higher in the third cycle in (cycle 2, August) compared with the third cycle in the other 22 districts (cycle 3, September; χ^2^ (df=1): 9.23, *p*=0.002), but significantly lower in the fourth cycle (χ^2^ (df=1): 28.16, *p*<0.002).

In addition, a significantly higher proportion of eligible children in Mangodara received Day 1 SPAQ in at least two, three or four cycles than in the remaining 22 districts.

### Qualitative results

Table 2 below outlines the sociodemographic characteristics of participants in the qualitative component of the study; caregivers, community distributors and key informants.

**Table 2:** Sociodemographic characteristics of qualitative study participants

Characteristic		Caregivers	CDs	KIs
Age (years)	Min Max Mean	21 58 35.4	22 48 30.79	33 72 50.90
Sex (M/F)	M F	20 (50%) 20 (50%)	22 (75.86%) 7 (24.14%)	10 (90.91%) 1 (9.09%
Religion	Catholic Protestant Muslim	5 (12.5%) 4 (10%) 31 (77%)	1 (3.45%) 0 (0%) 28 (96.55%)	5 (45.45%) 0 (0%) 6 (54.55%)
Education level	Primary (grades 1–6) Middle (grades 7–10) Secondary (grades 11–13)	6 (15%) 5 (12.5%) 0 (0%)	9 (31.03) 12 (41.38) 0 (0%)	0 (0%) 1 (9.09%) 1 (9.09%)
	Other (e.g. Bible or Quranic School)	1 (2.5%)	0 (0%)	

### Knowledge about the five cycle SMC campaign

Male and female caregivers knew the campaign began ‘earlier’, started ‘early compared to last year’s’ and that the campaign lasted for a longer time. Both men and women recognised that a cycle had been ‘added’, that the ‘number of passages has increased’ and that the drugs were distributed five times instead of four in previous years. Men and women associated the start of the campaign with the ‘beginning of the rainy season’ and women with the start of farming and field activities including ‘collecting shea nuts’ and ‘clearing fields’. CDs and supervisors also acknowledged the campaign started early, that the programme had ‘decided to add another month’ and comprised five instead of four cycles.

“The SMC drugs in past years were different from this year’s because in past years they were distributed four times, but this year it is like adding one more and it became like five, five times...” (Female caregiver, Faradjan)

“…another difference is that the number of passages has increased and that also has increased the health of the children here. The campaign was limited to four passages but this year I found that it reached five. That is the difference between this year’s and last year’s” (Male caregiver, Madiasso)

#### Reasons for the additional cycle

Both male and female caregivers recognised the campaign started earlier to coincide with the rainy season ‘which brings malaria’ and that ‘starting the campaign before the critical period’ will prevent the disease. Some female caregivers thought there had been a delay in distributing bed nets, and because of this ‘they distributed the medicines earlier to prevent the disease’. However, some men and women were unsure why the campaign started earlier.

Male and female CDs and supervisors recognised that the SMC campaign had started in June because that is when the rains start and mosquitoes tend to appear. For example, the group of male and female CDs mentioned that ‘malaria generally begins in the rainy season’ and the rainy season starts in June and ‘it’s also in that period that mosquitoes are active’. Male CDs also suggested the earlier start was because ‘in June the rain starts so there are mosquitoes’. Female CDs were of the view that because malaria ‘kills a lot of people every year’ and there are ‘a lot of cases of malaria’, the programme decided to adopt five cycles. In addition, the female CDs suggested the extra cycle was added to ‘determine if five cycles will cure malaria more than four cycles’ and to see if ‘cases of malaria would decrease compared to previous years’.

“In my opinion, last year they let the rainy season approach before starting the distribution. Maybe if they did this year, it was to eliminate malaria. In my opinion, that is why they started early to ensure that there is health, because it is the rainy season that brings malaria…” (Male caregiver, Faradjan)

“In my opinion, the campaign started early this year because malaria was much more threatening and the cases of malaria in children were very early” (Female caregiver, Faradjan)

### Feasibility of five cycles

#### Campaign coincides with and disrupts farming season

An overriding concern among caregivers was that the earlier start to the SMC campaign coincided with farming responsibilities, which was disruptive and meant that the CDs may not find people at home. Women and men described how they were ‘in a hurry to get to the field’ and ‘at that time many people care more about their fields’ and ‘prioritize their fields’ so the distributors ‘find nobody at home’. Men further explained that women are unable to wait at home for the CD because ‘it is a waste of time’ and if they do not work on the farm ‘you won’t have something to eat in the evening’. Further to this, some men mentioned that some ‘husbands do not care about the SMC campaign’ and expect their wives to join them in the fields. Because of this many suggested starting the campaign even earlier, in April or May, when men and women are available and ‘people are easily found at home’. The campaign starting in June affected the work of CDs too, who also have farming responsibilities. They explained how the month of June ‘coincides with our work in the fields’ and ‘we start farming works in April and in June we start sowing’. Some CDs mentioned that the campaign ‘delayed our farming job’ and that their fields were still ‘half covered with grasses’ by the time they could start work on them. Key informants also recognized that the SMC campaign happens ‘during the agricultural period’ or ‘the intense farming period’. They acknowledged that most CDs are farmers who may not be available during the distribution period ‘on constraint of being on their farms’.

“We cannot know everything about the difficulties related to their field work, but they may come to certain compounds and find no one since it is the field work period. This is a difficulty” (Female caregiver, Faradjan)

“In June here, frankly speaking, everyone is struggling because this is the basis of cultivation, everybody has to prepare his field at the moment…Once the rainy season starts, it’s complicated for CDs to find someone at home. That's why the CDs cannot find people in the concessions right now. That is different from ignorance. The period of caterpillars (caterpillars are eaten as food in some parts of Burkina) collection is also a period of difficulties. Personally, I also go to collect because such things feed me. When I get up in the morning, I go to my workplace, my wife as well, so the CDs may come at home without finding someone” (Male caregiver, Madiasso)

### Acceptability of five cycles

#### Benefits of earlier SPAQ administration

Female caregivers thought that starting the administration of SPAQ earlier than in previous campaigns had contributed to ‘improvement of our children’s health’ and had ‘really fought against malaria’; men also expressed how it had ‘brought us good health’ and had ‘given health to children’. Both men and women talked about fewer visits to the hospital because of malaria, noticed that fewer children were on intravenous fluids at hospital and there was decreased attendance at the dispensary due to malaria. Female caregivers also felt the campaign period was ‘well-chosen’ because it coincided with the first rains, which is when mosquitoes appear, and this allowed for ‘better prevention of malaria since the disease had not yet spread’. They compared it to last year when ‘the campaign started late’ when ‘we were already at the full rainy season and malaria had already contaminated the children’. CDs and supervisors shared many of the same views as caregivers; those children suffered less from malaria and they had seen the number of cases of ‘children’s malaria’ in the health centre decrease with the additional SMC cycle. They also compared the additional cycle to previous years, when children used to get malaria ‘before the SMC campaign began’. Key informants at national level also recognized that the earlier start to the campaign had contributed to fewer cases of malaria and fewer sick children. They also mentioned that there was ‘enthusiasm for SMC’ among the population and they had accepted the additional dose. One also stated that ‘sparing one episode of malaria for each child in a family’ was important and helped reduce the financial burden for families.

“This year it has been observed that the children who have benefited from the medicines do not have fever and the attendance of the dispensary due to malaria has strongly decreased. Then the SMC campaign has greatly contributed to reduce the high rate of consultations at the health facility” (Female caregiver, Faradjan)

“We’re comfortable. It has brought us good health. The way malaria made us suffer in the previous years, we saw that it didn’t make us suffer like other years. At least for us, it has brought us health” (Male caregiver, Faradjan)

#### Children’s tolerance to an additional cycle

Many male and female caregivers agreed that children could tolerate or ‘bear’ five cycles of SMC. Women explained that ‘children can handle them’ and ‘the campaign is not year-round, only one visit per month’ and men also noticed that ‘children can bear the five doses [cycles]’ because after taking the doses ‘they did not get sick anymore’. However, several men were concerned that the dosage was too high for children, suggesting ‘you may have to decrease the dosage’ and ‘instead of five times let them do four times’.

“At the beginning of the distribution of the drugs in June, my child took the first dose, it made it suffer. In fact, it vomited and had diarrhoea and fever too. But I continued to give the rest, and it felt good. So I can say that at the first dose, if you are not courageous enough, you cannot continue. Because it scares you. It knocked the child down (laughs), it knocked the child down” (Female caregiver, Madiasso)

“In fact, people talk too much, some say that the drugs get the children sick. Those who say that just witnessed cases of negative effects. Some who noticed the negative effects in their children even tell the drug distributors not to come to them” (Male caregiver, Madiasso)

#### Reactions at policy and programme level

Although the pilot study was ‘welcomed’ there seemed to be some apprehension among key informants at national level about implementing five cycles of SMC. This stemmed from the fact that the pilot deviates from current WHO recommendations of four cycles. Several informants mentioned ‘some apprehension’ in the technical community over resistance because children are ‘exposed to the drug for longer periods’, and any intervention that does not comply with the guidelines ‘may raise concerns’ about resistance in young children. Others emphasized that the ministry ‘operates according to WHO guidelines’ and cannot do anything differently until the WHO ‘formally authorises’ such a change.

“Exactly, because for the moment, the recommendation is four passages. That’s what I was saying earlier, the five passages are pilot and are designed as a study…Now, if we want to move to a large-scale intervention…almost all traditional partners will ask you first what WHO says about this or that?” (Key informant, NMCP)

“As I say it’s resistance to treatment also the fact that the WHO has not yet formally authorized that” (Key informant, NMCP)

#### Community distributor workload

Female CDs said the additional cycle did not impact on their workload. Instead they said distributing the drugs was ‘a lot more valuable’ than four days of work and they could continue with their other work afterwards. Their responses implied that they were willing to carry out the extra work and it ‘did not prove difficult’ because they valued the health of children and the village. Male CDs were more likely to say that the additional cycle of SMC had affected their work; they talked about how the cycle in June had increased their tasks, meant they couldn’t work in their fields, increased their expenses and debts. However, men also said that they were ‘proud of the SMC campaign’ that ‘health comes first’ and ‘we want the children to be healthy’. One group of male and female CDs seemed to share a different view, that the job ‘had increased but not the support’ and they talked about feeling ‘obliged’ to do the work even if it ‘takes most of your time’ and they had other responsibilities at the same time.

“What impact do four days of work have on us? What we are going to do through the distribution of the drugs will be a lot more valuable than those four days of work. It is better we spend the four days to dispense the drugs, and if we finish dispensing the drugs, we shall continue with our work later” (Female community distributor, Faradjan)

“It did not prove difficult because we want our village to be healthy. It did not prove to be a difficulty at our level. We want to achieve health” (Female community distributor, Faradjan)

“This increased our expenses, walking fatigue and difficulties. Because we left the work we were supposed to do in June for the June SMC campaign. And at that time we got into trouble, but we had to go and get fuel for the campaign. So we can say that this June cycle increased our task during the campaign; but thanks to the benefits of this additional June cycle, we are taking the courage to do the campaign in this month of June so that malaria can be reduced in our community” (Male community distributor, Madiasso)

### Views on future campaigns

#### Caregivers support continuation of five cycles

Men and women expressed obvious support for the SMC campaign and the extra cycle. Many comments indicated caregivers were ‘in favour’ and supportive of the five cycles and wanted the campaign to continue. They discussed having ‘seen the benefits’ or ‘advantages’ of five cycles and that these positive outcomes have led to ‘acceptance’ and ‘support’ of the government campaign. Some women even suggested they would be happy ‘even if the ministry decides to go to six phases’. Men also expressed support and said there was ‘no difficulty’ or problem with this year’s campaign, and they would participate if it continues because the five cycles ‘have brought good health to our children’.

A few women and men mentioned the need ‘to know more about’ the drugs and be able to ask questions before understanding and accepting SMC; some were concerned about the side effects and ‘the fact that it weakens’ children. Others suggested a need ‘to find a way to raise awareness’ to increase people’s knowledge of the side effects and difficulties that can arise, because ‘it sometimes confuses people’ and ‘it freaks people out’. Some comments suggested that even if some in the community didn’t want to support the initiative, the government ‘knows about the number of sick children’ and ‘knows why’ they give medicines, so they will ‘go along with them’.

“We will agree with the Ministry because the SMC campaign was very successful. Given that, even if the Ministry decides to go to six phases, we're ready” (Female caregiver, Faradjan)

“We are going to support them because we have seen the benefits of the five cycles. They helped us and if it continues it is good for us” (Female caregiver, Madiasso)

“We are going to agree because there are advantages. We also see the benefits; that is why we are going to support them” (Female caregiver, Madiasso)

#### Community distributors face many difficulties but are driven to improve health of the village

An important overarching concern among CDs was that they faced numerous difficulties in delivering the SMC campaign; because of this they felt ‘discouraged’ and the problems were significant enough for many to consider ‘giving up working on it’ in the future. An important challenge raised by most CDs was the delay in remuneration for their drug distribution work, which often left them out of pocket for significant amounts of time. Some requested to be paid more quickly after the campaign and complained that the conditions this year meant they worked for three cycles and only received payment for the first month, or that the only payment they had received early was for training. Another challenge was sourcing fuel to travel to distant farming villages for drug distribution; many talked about having to borrow fuel or obtain it on credit, others mentioned feeling ‘disgraced’ spending months without being able to repay creditors. Others talked about having to borrow motorbikes ‘to perform the campaign’ and wanted transportation to be provided. Male CDs felt that supervisors did not ‘know the reality’ of having to visit ‘farming hamlets’ and emphasised that if working conditions remained the same in coming years, they ‘will not be able to do it’, ‘we will resign’ and if there is no change in conditions ‘there is no need to call us for the coming years’. Despite these challenges dominating the discussions, most groups of CDs were quick to acknowledge the importance of the fifth cycle and getting SMC drugs to children, and affirmed that the health of children is what ‘encourages us to continue with the job’. Most said they were prepared and willing to do the job and the earlier start date was not problematic; rather the campaign needed to ‘manage the situation’ and ‘solve the difficulties”.

“Another issue is the means of transportation. An important additional issue is the financial one. We actually appreciate the job because it is important for our community to get the medicines and also it is true that is it for our children but we the distributors are all farmers…But sometimes we finish the whole job without being paid. By the time you get paid, you are obliged to sell a part of your harvests in order to pay your employees. This is also a serious issue” (Female community distributor, Madiasso)

“If they could improve the conditions, it would be good for us because we like the work. We don't want the conditions of this year. We have done three cycles and we only got the compensation of the first month. When it is like that, it is as if you had no compensation. Because it makes us lazy. Previous years, on the same night the campaign ended, we would sign a paper and take our money and we could pay the petrol credit; but this year it is not the case” (Male community distributor, Madiasso)

### Key informants concerned about the need for more evidence

Many national level key informants said they would recommend the fifth cycle, but not without ‘scientific evidence’ or until it is ‘scientifically proven’. Many discussed that the five cycles may not be needed everywhere, and a targeted approach might work, implementing where there is a good chance of ‘maximising the impact’; one suggested a situation analysis to detect ‘peak transmission’ areas that would benefit most.

Several were appreciative of the pilot study and Malaria Consortium for helping provide ‘some answers’ and evidence on whether the fifth cycle is needed, to guide on the next steps. Key informants also realised that for sustainability there would need to be continuous advocacy and sensitisation at community level to ‘get their attention’ and ‘explain to people the reasons for an additional visit’; especially information for mothers before each round, which they thought would be the ‘cornerstone’ of the campaign.

“I will say yes because if there is real value in that, why not? Because we are all, we are all working to reduce the burden (drawbacks) of malaria. It is an intervention that we are sure can reduce the rate of malaria so we can recommend it. Now what I’m saying, recommending it doesn’t mean doing it systematically everywhere. Maybe we can see where we have the best chance of maximizing the impact of this intervention. As I was saying, in the Sahel it may not be necessary; it may not be necessary to do five interventions” (Key informant, Jhpiego)

“So the problem is not, about acceptability or not, it’s a problem of scientific evidence, and for us to actually align ourselves with it.” (Key informant, Global Fund)

“the additional cycle was more or less desired for specific areas. I think that’s how I understood it. Areas are not the same in Burkina. There are areas where the rainfall starts a little earlier than the others. SMC being this strategy which allows to prevent malaria during the peak transmission periods, a situation analysis throughout the country is necessary, considering the different area, in order to see roughly when these peak transmission periods occur” (Key informant, Banfora city)

## Discussion

The primary aim of this study was to evaluate whether extending SMC from four to five cycles was feasible and acceptable. An additional cycle of SMC was distributed to children under five in June 2019 in Mangodara district, Burkina Faso. All 22 remaining eligible districts supported by Malaria Consortium received four cycles of SMC from July to October 2019. SMC has been extended beyond the WHO-recommended four-cycle regimen in previous studies [14, 16], and the acceptability of five cycles has been described in a study in Ghana which primarily focussed on the barriers and facilitators to uptake of four or more doses [16]. This is the first study to explore both the acceptability and feasibility of implementing five cycles of SMC across an entire district under programmatic conditions in Burkina Faso.

The general consensus was that five cycles of SMC were broadly acceptable to caregivers and community distributors because of the enhanced health protection offered to children, contributing to fewer cases of malaria and fewer sick children. This is in line with findings from a similar study where the length of the five-month SMC programme was acceptable to caregivers [16].

Our results show more than 87% coverage of SMC across all cycles in Mangodara and an increase in coverage from the first to fifth cycle. Furthermore, the proportion of eligible children covered in cycle 0 in Mangodara compared to subsequent cycles was slightly less; this trend was similar in the other 22 districts, with coverage lower in cycle 1 than in cycles 2–4. This could be due to poorer organisation of SMC delivery earlier on in the campaign or recall bias, i.e. caregivers may be more likely to remember more recent cycles. Although the difference was small, the results of the chi-squared tests showed this was statistically significant. The addition of a fifth cycle in Mangodara was found to have significantly increased the proportion of children who had received four cycles of SMC.

Caregivers, community distributors and stakeholders perceived the fifth cycle of SMC to reduce the cases of malaria among children under five and caregivers’ expenses for transporting children to, and receiving treatment at, health facilities through less frequent visits. This high level of community acceptance was recognised by national-level key informants who observed ‘enthusiasm for SMC’. Evidence suggests that community acceptance of malaria mass drug distribution campaigns is largely due to intensive sensitisation and mobilisation [21]. Information provided at the beginning of the SMC campaign by town criers allowed most of the participants to identify and understand the difference between previous SMC campaigns and the 2019 campaign, which aided acceptability. Our findings suggest that effective community sensitisation, coupled with the desire for the health for their children, may motivate caregivers to participate in future five-cycle SMC campaigns.

Coverage was significantly higher in Mangodara compared to the 22 other districts based on statistical tests (when comparing coverage in the same month), however, coverage was high across all cycles in both. The slight difference in coverage between Mangodara and the other districts is likely to be unimportant from a programmatic perspective, and it is likely that our results could be generalisable to other districts in Burkina Faso. However, other social factors such as local knowledge, attitudes and practices related to SMC and other social determinants must be taken into consideration prior to geographical expansion.

However, despite the fifth SMC cycle being positively appreciated by all participants, many community distributors expressed concern over the feasibility, mainly due to the clash with farming activities in June. This meant some caregivers were not available to receive SMC and delayed some community distributors in completing their responsibilities. A potential solution could be to introduce community-led initiatives, such as appointing the chief of the village to choose heads of households to work on the farms in place of the community distributor in June, in addition to sensitisation and awareness-raising around the additional cycle, to encourage caregivers to be available. Alternatively, community distributors must visit each compound early in the morning before parents and their children leave for the field. However, the average time taken to administer SPAQ per child and the number of compounds that can be feasibly visited early in the morning must be determined prior to implementation of this approach. Increasing the number of community distributors may also provide a viable solution, however would incur an additional cost for national SMC programmes which would require further exploration.

Furthermore, male caregivers described how some ‘husbands do not care about the SMC campaign’ and expect their wives to join them in the fields. Therefore, a concerted effort must be made to actively engage male caregivers in awareness-raising and communication sensitisation activities prior to the start of each campaign to improve their knowledge of SMC, including the importance of the intervention and their participation. In addition to the clash with farming activities, male community distributors emphasised that the additional SMC cycle impacted on their workload and increased their expenses and debts. This was particularly problematic as the level of support did not also increase in proportion to the workload, leaving them feeling ‘discouraged’. Should a fifth SMC cycle be implemented in the future, remuneration must be paid on time and in the correct amount as currently, this is often late or insufficient (e.g. only for the first cycle).

Whilst many national-level key informants recommended the fifth cycle, the results suggest they were more conservative about their support than community distributors and caregivers. Their primary concern was that more evidence is needed to demonstrate the impact of five cycles and the development of resistance. A recent study that collected data on molecular markers of resistance to SPAQ across West Africa showed that these remained uncommon, however there is a need to carefully monitor this through the setup of surveillance systems to inform a tailored approach [9].

Overall, stakeholders at the community, regional and national level accepted five cycles of SMC delivery due to its perceived positive health impact on children under five. However, there are critical challenges and key recommendations which need to be addressed to make the five-cycle campaign more feasible. More evidence is needed on the impact of the extended campaign on malaria incidence and parasite resistance prior to scaling up. Also, as rainfall patterns are irregular across the country, it will be important to highlight the areas where fewer or more than four cycles of SMC are needed.

## Limitations

This study had a few limitations. Due to a country-wide strike by community distributors during the 2019 campaign, Malaria Consortium were unable to access Health Management Information System data and conduct an analysis into how the additional cycle affected malaria incidence in Mangodara. Tally sheet data were also unavailable due to the strike, preventing the estimation of administrative coverage, i.e. the number of SPAQ doses administered per cycle. The paper cannot account for contextual factors that may explain the statistical difference between the coverage of SMC in Mangodara compared to the other districts as there was no randomisation of clusters within districts to receive the additional cycle. The surveys were adjusted to allow for the analysis of coverage in the extra cycle after Mangodara district had been selected. Heads of households were underrepresented in the caregiver FGDs due to competing priorities (gold-mining duties 50km away) in the village of Faradjan. As the most influential and powerful member of the household, their perspective of the extension is important as they have the decision-making power on whether their child will receive an additional cycle of SMC. It was challenging to recruit a sufficient number of female CDs to participate in FGDs on their own, resulting in more mixed FGDs than anticipated. The views of female CDs may therefore be underrepresented in these results. Furthermore, participants may have been subject to social desirability and self-report bias during FGDs and KIIs, potentially impacting on the reliability and validity of data. Due to challenges in scheduling the FGDs and KIIs, there was a one-month delay from the end of the 2019 campaign in October to when the data were collected, increasing the potential for recall bias.

## Conclusions and recommendations

Extending SMC from four to five cycles is perceived as acceptable by caregivers, community distributors and stakeholders, and to have a positive effect on the health of children aged under five. Community distributors are motivated to support a fifth cycle and this has been demonstrated by a high coverage of SMC in Mangodara in all five cycles. The fifth cycle of SMC distribution should begin early in the day in order to not coincide with the farming activities of community distributors, who are mainly farmers. However, the average time taken to administer SPAQ per child and the number of compounds that can be feasibly visited early in the morning must be determined beforehand. Particularly as the additional cycle will increase community distributors’ workload, there is a need to improve their motivation by avoiding late payments and providing fuel and a mode of transportation. Finally, continuous advocacy and sensitisation at the community level are critical for the sustainability of SMC and acceptance of an additional cycle. Further studies are required to understand the effectiveness, including cost-effectiveness, of varying the number of SMC cycles according to the timing and duration of the rainy season.

## Data Availability

The datasets used and analysed during the study are available from the corresponding author on reasonable request.

## Abbreviations

AQ: Amodiaquine
CD: Community distributor
EoC: End-of-cycle
EoR: End-of-round
FGD: Focus group discussion
KII: Key informant interview
NMCP: National Malaria Control Programme
SMC: Seasonal malaria chemoprevention
SP: Sulfadoxine-pyrimethamine
WHO: World Health Organization
